# Microembolus clearance through angiophagy is an auxiliary mechanism preserving tissue perfusion in the rat brain

**DOI:** 10.1186/s40478-020-01071-9

**Published:** 2020-11-17

**Authors:** Anne-Eva van der Wijk, Theodosia Georgakopoulou, Jisca Majolée, Jan S. M. van Bezu, Miesje M. van der Stoel, Bert J. van het Hof, Helga E. de Vries, Stephan Huveneers, Peter L. Hordijk, Erik N. T. P. Bakker, Ed van Bavel

**Affiliations:** 1 Amsterdam UMC, Biomedical Engineering and Physics, Amsterdam Cardiovascular Sciences, University of Amsterdam, Meibergdreef 9, Amsterdam, The Netherlands; 2 Amsterdam UMC, Amsterdam Cardiovascular Sciences, Physiology, Vrije Universiteit Amsterdam, De Boelelaan, 1117 Amsterdam, The Netherlands; 3 Amsterdam UMC, Medical Biochemistry, Amsterdam Cardiovascular Sciences, University of Amsterdam, Meibergdreef 9, Amsterdam, The Netherlands; 4 Amsterdam UMC, Molecular Cell Biology and Immunology, Amsterdam Neuroscience, Vrije Universiteit Amsterdam, De Boelelaan, 1117 Amsterdam, The Netherlands; 5 Department of Biomedical Engineering and Physics, Academic Medical Center, Room L0-120, 1100 DD Amsterdam, The Netherlands

**Keywords:** Angiophagy, Cerebral microcirculation, Embolus, Endothelial cells

## Abstract

Considering its intolerance to ischemia, it is of critical importance for the brain to efficiently process microvascular occlusions and maintain tissue perfusion. In addition to collateral microvascular flow and enzymatic degradation of emboli, the endothelium has the potential to engulf microparticles and thereby recanalize the vessel, through a process called angiophagy. Here, we set out to study the dynamics of angiophagy in relation to cytoskeletal remodeling in vitro and reperfusion in vivo. We show that polystyrene microspheres and fibrin clots are actively taken up by (brain) endothelial cells in vitro, and chart the dynamics of the actin cytoskeleton during this process using live cell imaging. Whereas microspheres were taken up through the formation of a cup structure by the apical endothelial membrane, fibrin clots were completely engulfed by the cells, marked by dense F-actin accumulation surrounding the clot. Both microspheres and fibrin clots were retained in the endothelial cells. Notably, fibrin clots were not degraded intracellularly. Using an in vivo microembolization rat model, in which microparticles are injected into the common carotid artery, we found that microspheres are transported by the endothelium from the microvasculature into the brain parenchyma. Microembolization with microspheres caused temporal opening of the blood–brain barrier and vascular nonperfusion, followed by microsphere extravasation and restoration of vessel perfusion over time. Taken together, angiophagy is accompanied by active cytoskeletal remodeling of the endothelium, and is an effective mechanism to restore perfusion of the occluded microvasculature in vivo.

**Electronic supplementary material:**

The online version of this article (10.1186/s40478-020-01071-9) contains supplementary material, which is available to authorized users.

## Introduction

Although the brain comprises only 2% of the body weight, it is responsible for approximately 20% of the total body’s energy expenditure [24]. Since the brain has no substantial local energy storage, it has a critical need for a constant cerebral blood flow. In order to maintain sufficient perfusion to meet its energy demands, the brain has a uniquely organized vascular network. The cerebral cortex is supplied and drained by interconnected pial arteriolar and venular networks, connected through penetrating arterioles and venules with the three-dimensional microvascular network feeding the parenchyma [2]. This intricate microvascular network ensures tissue perfusion throughout the brain, with every neuron positioned within < 15 µm from a capillary [26].

Obstruction of the cerebral microvasculature impairs local perfusion and leads to ischemic damage. Such obstruction can result from brain embolization, with microemboli originating from various sources. For instance, patients with atrial fibrillation have a substantial burden of vascular brain lesions [6], of which a large portion may be caused by cardioembolic events. Although many of the lesions are clinically unnoticed, they are associated with decreased cognitive function [6]. Recently, it became clear that (micro)embolization of the brain may be a major complication of the infectious disease COVID-19 [15]. Furthermore, the introduction of intra-arterial thrombectomy in the treatment of acute ischemic stroke has surfaced a major challenge in the field. Recanalization of the occluded artery is achieved in the large majority of patients after thrombectomy, yet the procedure itself may result in emboli showers dislodging from the main thrombus [4, 5, 29], causing widespread microvascular perfusion deficits distal of the initial event [29].

Microembolus composition and location determine the extent of tissue damage caused by an occlusion. Microemboli may be rich in red blood cells, have different characteristics depending on thickness and density of fibrin strands [9], or consist mainly of cholesterol [19], calcified material [20] or white blood cells [7]. This specific composition makes clots more or less prone to degradation, hence affecting the resolution of tissue ischemia. In addition, the extent of collateralization of the vascular plexus where the occlusion or embolus occurs determines whether alternative pathways for perfusion are available [22, 23] or not [18, 23], and thus, whether local tissue perfusion is maintained.

Considering its intolerance to ischemia, it is very important for the brain to efficiently process small emboli and maintain tissue homeostasis. Although the three-dimensional microvasculature is highly interconnected, collateral microvascular flow is not always sufficient to maintain tissue perfusion. Whereas the brain has endogenous fibrinolytic capacity [25, 29], many emboli are poorly susceptible to this enzymatic degradation [17], with prolonged vessel occlusion and hypoxia as a result. Notably, the endothelium has the potential to engulf microparticles from the circulation and extrude them on the abluminal side, in a process called angiophagy. This extravasation of microparticles was found in the brain and other organs and with emboli of different sources in mice [12, 16], rats [27] and in the human retina [3, 12]. Crucially, angiophagy may be a protective mechanism of the brain to reestablish blood flow through vessel recanalization [16].

Here, we show that microparticle uptake is associated with active cytoskeletal remodeling of endothelial cells in vitro, using fibrin clots and polystyrene microspheres. Furthermore, we quantified the percentage of perfused vessels after microembolization and demonstrate that embolus extravasation in vivo, or angiophagy, is needed to restore vessel perfusion in a microembolization model in rats.

## Methods

### Cell culture and in vitro endothelial uptake experiments

Human umbilical vein endothelial cells (HUVECs; Lonza, Verviers, Belgium, passage 2–5) were grown to confluence on 5 µg/ml fibronectin-coated glass coverslips (Menzel™, ThermoFisher, Amsterdam, The Netherlands) and kept in Endothelial Cell Medium (ECM) supplemented with singlequots (ScienCell Research Laboratories, Carlsbad, CA) at 5% CO_2_ at 37 °C. Human cerebral microvascular endothelial cells (hCMEC/D3, passage 29-31) were grown in type I collagen-coated Ibidi slides in EGM™-2 BulletKit™ Medium (Lonza). Microspheres (15 µm; 10,000 per well (surface area 1.9 cm^2^)) or Texas Red-conjugated fibrin clots (~ 2700 per well (surface area 1.9 cm^2^); see Additional file 1: Supplementary Materials and Methods), were added to the cells in a volume of 50 µl. Based on approximate cell numbers in confluent HUVEC cultures and added microspheres, this is 1 microsphere per ~ 10 cells. After 4 or 24 h cells were fixed with 4% paraformaldehyde for 15 min and immunofluorescence staining was done (Additional file 1: Supplementary Materials and Methods).

Images were recorded using a confocal laser scanning microscope SP8 (Leica Microsystems, Wetzlar, Germany) with a 63 × 1.40 NA oil objective. Microparticles were imaged at five random locations per well and z-stacks were made based on phalloidin signal with z-steps of 0.30 µm. Images were deconvolved using Huygens Professional version 19.04 (Scientific Volume Imaging, The Netherlands, http://svi.nl) and 3D images were rendered using LAS X 3D software (version 3.7.0; Leica Microsystems).

### Lentiviral transductions and live cell imaging

Lentiviral particles were generated by transfecting HEK293T cells with the lentiviral expression construct with 3rd generation packaging plasmids using *Trans*IT^®^-LT1 transfection reagents (Mirus Bio LLC, Madison, WI). The supernatant containing the lentiviral particles was harvested 48-72 h post transfection. HUVECs were transduced with LifeAct-mTurquoise construct (as described in Goedhart et al. 2012 [11]) cloned into a Lentiviral vector (pLV) overnight, selected with puromycin and grown to confluence on fibronectin-coated Lab-Tek Chambered 1.0 borosilicate coverglass slides (ThermoFisher) in EGM™-2 medium (Lonza). Live cell imaging was done with an inverted NIKON Eclipse Ti equipped with a 60 × 1.49 NA Apo TIRF (oil) objective, perfect focus system, CFP and mCherry filter cubes and an Andor Zyla 4.2 plus sCMOS camera. An Okolab cage incubator and humidified CO_2_ gas chamber set to 37 °C and 5% CO_2_ were used during imaging. Frames were taken every minute for 4–8 h.

### Animals

The animal experiments in this study were conducted in female and male Wistar rats (16 to 20 weeks old, Charles River). Rats were housed in pairs under a 12 h light–dark cycle and fed ad libitum with standard laboratory chow and free access to water. All surgical procedures were conducted under isoflurane inhalation anesthesia mixed with oxygen while the body temperature was monitored with a feedback-regulated heating pad. Adequate depth of anesthesia was verified by absence of toe-pinch reflex.

### Cerebral embolization procedure

On the day of surgery, animals were weighed and anesthetized with isoflurane (induction 4%, maintenance 2–2.5% in 1 L/min O_2_). Buprenorphine (0.05 mg/kg) was administered subcutaneously 30 min prior to the first incision for analgesia. The rat was placed in a supine position on a homeostatic heating pad, hair was removed from the skin caudal to the mandibles and iodide was used to disinfect the skin. A midline incision was made from below the mandible to the sternal notch. To expose the left carotid artery around the bifurcation, the superficial fascia, salivary glands and the muscle layers were blunt dissected. The left common carotid (CCA), the internal carotid (ICA) and external carotid (ECA) artery were exposed and the ECA and occipital artery were temporarily ligated with 6–0 surgical sutures. Next, a mixture of fluorescent microspheres (DiagPoly™ Plain Fluorescent Polystyrene Particles, λ Ex 530 nm, λ Em 582 nm, Creative Diagnostics^®^, Shirley, NY) or fibrin clots (10,000 particles; Additional file 1: Supplementary Materials and Methods; Additional file 2: Fig. S1) was injected into the left CCA using a 29 G insulin needle (25,000 of 15 µm (14.1 ± 1.2), 5500 of 25 µm (26.3 ± 3.0) and 625 of 50 µm (50.4 ± 3.3) microspheres, resuspended in sterile 2% bovine serum albumin in phosphate buffered saline (PBS), in a total volume of 200 µl). No microparticles were injected in the right CCA, and therefore the right hemisphere served as the untreated control. To stop the bleeding after removal of the needle, pressure was applied to the injection site. Subsequently, threads were removed. The wound was closed with sutures and rats were allowed to recover on a heating pad before returning to their cages. One animal had to be killed directly after the surgery because it did not recover well from anesthesia and was excluded from analysis. Two sham animals, in which the ECA and occipital artery were ligated and 2% bovine serum albumin in PBS was injected in the CCA, were killed 1 day after surgery to serve as a control for surgery and anesthesia effects on edema and IgG extravasation but not included in the final analysis.

### Tissue preparation and immunofluorescence staining

Animals injected with microspheres were killed on day (D) 1 (n = 8), 3 (n = 8) or 7 (n = 8) after surgery, and animals injected with fibrin clots were killed on D1 (n = 4) or within an hour (n = 1) after surgery. After induction of anesthesia, the vasculature was labeled by an intravenous injection of DyLight 594 labeled tomato lectin (1 mg/kg; DyLight 594 labeled lycopersicon Esculentum tomato, Vector Laboratories, Burlingame, CA) and allowed to circulate for 5 min. Rats were given 100 µl heparin i.p. and after increasing isoflurane to 5% animals were transcardially perfused with heparinized PBS followed by tissue fixation with 4% paraformaldehyde at 80 mmHg. Tissue preparation and immunofluorescence staining are further described in Additional file 1: Supplementary Materials and Methods. Images of brain sections were captured using a confocal laser scanning microscope SP8 (Leica Microsystems) with a 10× 0.5 NA (air) or 20× 0.75 NA (oil) objective.

### Image analysis

Five animals were excluded from analysis because the microsphere injections were not successful (i.e. no or very few microspheres were found in the brain—both vessels and parenchyma), resulting in the analyses being done on D1 (n = 6), D3 (n = 6) and D7 (n = 7). Quantification of microsphere extravasation was performed on sections stained for laminin. Microsphere extravasation was scored as “in” (microspheres are inside a vessel), “going out”/extraluminal (in the process of extravasation, in many cases observed as a vessel with lectin perfusion yet with the microsphere still contained within the extracellular matrix, often seen as a laminin bulge); or “out”/parenchymal (outside of the vessel lumen and the extracellular matrix) by two independent researchers blinded to the identity of the specimen. An extravasation score was calculated for each animal to enable statistical analysis, where microspheres scored as “in” were given 0, “going out” 1 and “out” 2. Vessel nonperfusion was quantified in the same samples, and a vessel was identified as “nonperfused” when there was no lectin distal and/or proximal from the occluding microsphere. Midline shift was calculated by measuring the width of the control and treated hemispheres starting from the midline, and divided by the total width from ex vivo coronal sections in FIJI (version 2.0.0-rc-69/1.52 m). Three sections were measured and averaged per animal. IgG leakage, GFAP and Iba1 signal intensity was assessed by determining mean pixel intensity in overview images, while excluding ventricles using threshold analysis in FIJI (all pixels with “0” were excluded). All analyses on animal brain sections were quantified in a blinded fashion.

### Statistics

Data are depicted as median ± interquartile range (IQR: box; and whiskers: min–max) or mean ± standard deviation (SD). Data was tested for normality using a QQ-plot and a Shapiro–Wilk test and depending on the outcome, a parametric or nonparametric test was performed. Differences between treated and untreated hemispheres were determined using a Wilcoxon signed rank or paired *t* test. Differences between groups (D1, 3 and 7) were determined using analysis of variance (ANOVA) followed by Tukey–Kramer’s test for multiple comparisons, or by Kruskal–Wallis with Dunn’s test for multiple comparisons. Between-size (15 µm and 25 µm microspheres) differences were tested with a repeated measures 2-way ANOVA and association of categorical variables was tested with a Chi square test. Differences were considered statistically significant when *P* ≤ 0.05. Statistical analyses and graphing were performed using GraphPad Prism 6 software (GraphPad Software, La Jolla, CA).

## Results

### Microparticles are taken up by endothelial cells in vitro

**Fig. 1:**
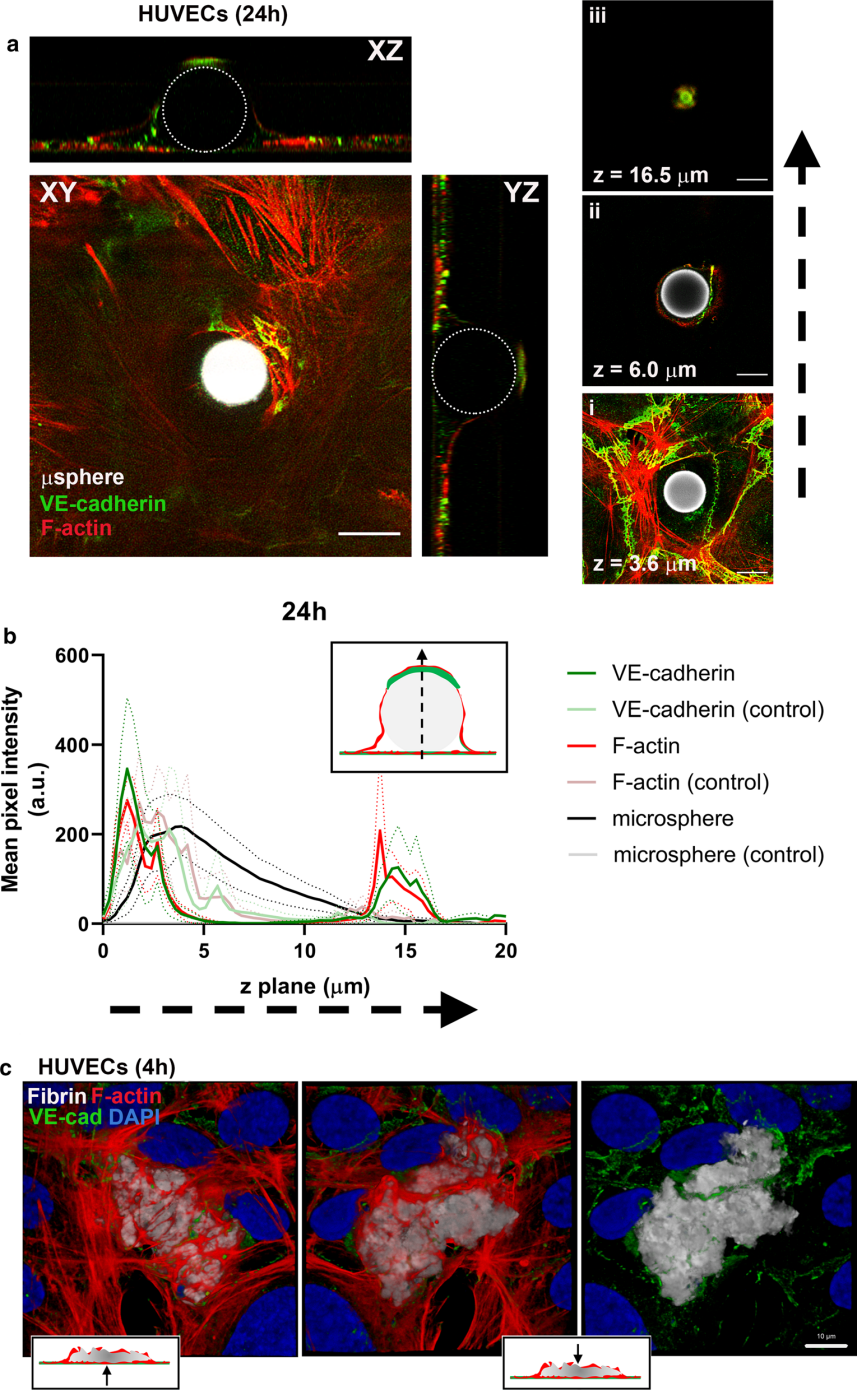
Microspheres are taken up by HUVECs. **a** XZ and YZ orthogonal view of a z stack shows a cup structure of phalloidin (F-actin; red) surrounding the microsphere (white; in XZ and YZ depicted with dashed line), and F-actin- and VE-cadherin-positive “caps” on top of the microsphere. Different z planes are shown in i, ii and iii. Note the F-actin and VE-cadherin (green) surrounding the microsphere in ii, and the cap on top of the microsphere in iii. Scale bar = 10 µm. **b** Quantification of signal intensity for F-actin, VE-cadherin and microsphere (dark-colored lines) in the z direction shows a peak in signal intensity after z = 15 µm, which is the cap structure on top of the microsphere. Light-colored lines are signal intensity in control location, i.e. of a region where no microsphere was bound. Signal intensity was quantified from 2 to 4 images averaged from n = 3 independent experiments. Data are depicted as mean ± S.D. (dashed lines). **c** Three-dimensional rendering of a fibrin clot, encapsulated by the cytoskeleton with intact adherens junctions. Left panel is the view from below the cellular monolayer, middle panel is the view from above the monolayer. Right panel shows that the fibrin clot is taken up by two cells, demonstrated by the intact adherens junction between cells across the clot. Cell nuclei are blue (DAPI). Scale bar = 10 µm

To determine whether microparticles are taken up by endothelial cells in vitro, we added microspheres (15 µm) and fibrin clots (10–40 µm) to HUVEC monolayers followed by fixation after 4 and 24 h. Already after 4 h, we observed that the endothelial cells generate apical membrane protrusions, forming cups around the microspheres. In all cases, F-actin- and VE-cadherin-positive “caps” were observed on top of the microspheres in addition to F-actin in the basal plane of the cup structures (Fig. 1a, Additional file 3: Video S1), indicating that the microspheres were completely engulfed by the endothelial cell. In Fig. 1b, we quantified the fluorescence profile for the adherens junction protein VE-cadherin and F-actin in the z direction, demonstrating location of these caps at + 15 µm relative to the apical membrane. Cell–cell contacts remained intact despite uptake of microspheres, as deduced from the VE-cadherin signal at intercellular junctions (Additional file 3: Video S1). In addition, we observed that fibrin clots in many cases were completely internalized by HUVECs already after 4 h, with dense F-actin concentrations in the apical membrane (Fig. 1c, Additional file 3: Video S1). In some cases, this was a joint effort performed by multiple cells, as shown by intact adherens junctions between cells engulfing the clot (Fig. 1c). HUVECs are endothelial cells of peripheral, macrovascular origin, and may respond differently to stimuli compared to barrier-forming, microvascular endothelium of the central nervous system [28]. Therefore, we also used hCMEC/D3 cells, often used as a simplified model of the human blood–brain barrier [30] (BBB). Similar to HUVECs, endothelial membrane protrusions and “caps” were found surrounding the microspheres (Additional file 4: Fig. S2A, B), and fibrin clots were encapsulated by F-actin rich membrane protrusions (Additional file 4: Fig. S2C).

### Engulfed microparticles are retained intracellularly in live cells

Using a lentiviral approach, we transduced HUVECs with LifeAct-mTurquoise to visualize the F-actin cytoskeleton dynamics during microparticle uptake in live cells. Similar to what we observed with immunofluorescence staining, addition of microspheres to the cells induced the formation of an endothelial apical, F-actin rich cup, surrounding the microsphere (Additional file 5: Video 1). This cup generally formed within 2–4 h after microsphere addition, and microspheres were retained by the endothelial cell that had taken up the particle, even during cell locomotion and division. For fibrin clots, we observed F-actin-rich points of contact, taking hold of the clot at multiple sites, and F-actin accumulation around the clot (indicated with open arrow in Additional file 6: Video 2). Once taken up inside the cell, we consistently observed an imprint of the fibrin clot in the F-actin cytoskeleton (indicated with filled arrow in Additional file 6: Video 2). Of note, fibrin clots were not degraded intracellularly, but were carried along by the cell that had taken up the particle, similar to the microspheres. Taken together, these in vitro data show that microparticles are retained intracellularly once they are taken up and fibrin clots are not degraded by endothelial cells.

### Microparticles do not affect endothelial barrier function in vitro

**Fig. 2:**
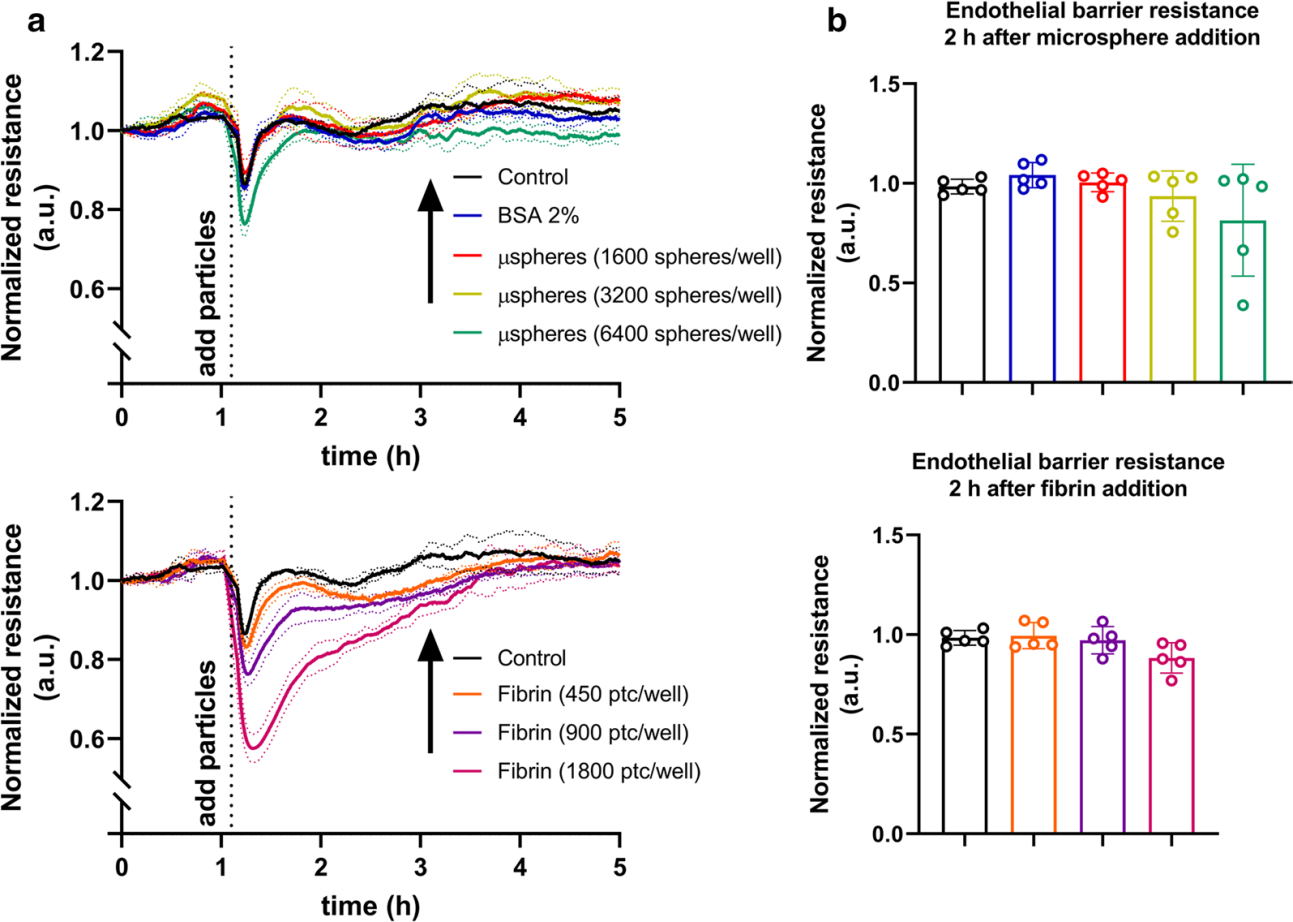
Impedance measurements with ECIS after addition of microparticles to HUVECs. **a** Example tracing of an ECIS experiment after addition of different amounts of microspheres or fibrin clots to endothelial cells. Tracings are shown as normalized electrical resistance, where t_0_ is the baseline electrical resistance of ~ 1 h prior to addition of microparticles. Baseline electrical resistance at 400 Hz was 1086 Ω (median; range 633–1513 Ω). The arrows indicate the time point used for quantification in **b**. **b** Quantification of electrical resistance at 400 Hz 2 h after addition of microparticles. N = 5 independent experiments done in triplo. Data are depicted as mean ± S.D. One-way ANOVA with Tukey–Kramer’s test for multiple comparisons

We assessed possible endothelial barrier loss by microparticles using Electric Cell-Substrate Impedance Sensing (ECIS). We found that the addition of microspheres and fibrin clots did not alter electrical resistance of HUVEC monolayers (Fig. 2a, b), except for 2 of the 5 experiments where endothelial barrier function was negatively influenced after addition of the highest amount of microspheres (green bar, Fig. 2b). However, this amount exceeded the amount used in the experiments Fig. 1, which corresponds to the red bar in Fig. 2b. To test for preservation of endothelial cell responses, we used a thrombin receptor activating peptide 24 h after the addition of microparticles, which caused a rapid drop and recovery of electrical resistance similar to that seen in control cells (data not shown). This suggests that microparticles did not affect endothelial reactivity or viability.

### Timing of microsphere extravasation in vivo

In our previous work, we established that microspheres (15 µm) can extravasate from the vessel lumen in the rat cerebral microcirculation [27]. Here, we injected a mixture of different microsphere sizes (15, 25 and 50 µm) into the internal carotid artery of rats to determine whether there is a size limit for emboli to undergo extravasation and if size affects extravasation rate. In Additional file 1: Supplementary Table S1, percentages of microspheres injected and detected in the brain are given. We only occasionally observed 50 µm microspheres in the brain parenchymal vessels. We injected 625 50 µm microspheres per animal as to not induce severe neurological damage, and it is possible that a large portion of these microspheres remained in the pial vasculature as was demonstrated previously with magnetic resonance imaging [31], and that we were unable to detect them in our coronal sections. Because of the low number of observed 50 µm microspheres, we limited our analysis to the smaller microspheres.

**Table 1:** Nonperfusion of vessels is related to extravasation status

	Perfused (#)	Nonperfused (#)	Total scored (#)	Nonperfused (%)	*P*-value (X^2^)
**Day 1 (n = 6)**					
15 µm					
In	40	120	160	75.0	0.0016 (**)
Going out	5	1	6	16.7	
Out	0	0	0	n/a	
25 µm					
In	5	26	31	83.9	0.6620 (ns)
Going out	0	1	1	100	
Out	0	0	0	n/a	
**Day 3 (n = 5)**					
15 µm					
In	79	83	162	51.2	0.0013 (**)
Going out	9	6	15	40.0	
Out	11	0	11	0.0	
25 µm					
In	9	21	30	70.0	0.0242 (*)
Going out	3	2	5	40.0	
Out	2	0	2	0.0	
**Day 7 (n = 6)**					
15 µm					
In	24	76	100	76.0	< 0.0001 (****)
Going out	25	4	29	13.8	
Out	62	4	66	6.1	
25 µm					
In	7	13	20	65.0	0.0003 (***)
Going out	18	6	24	25.0	
Out	9	0	9	0.0	

**Fig. 3:**
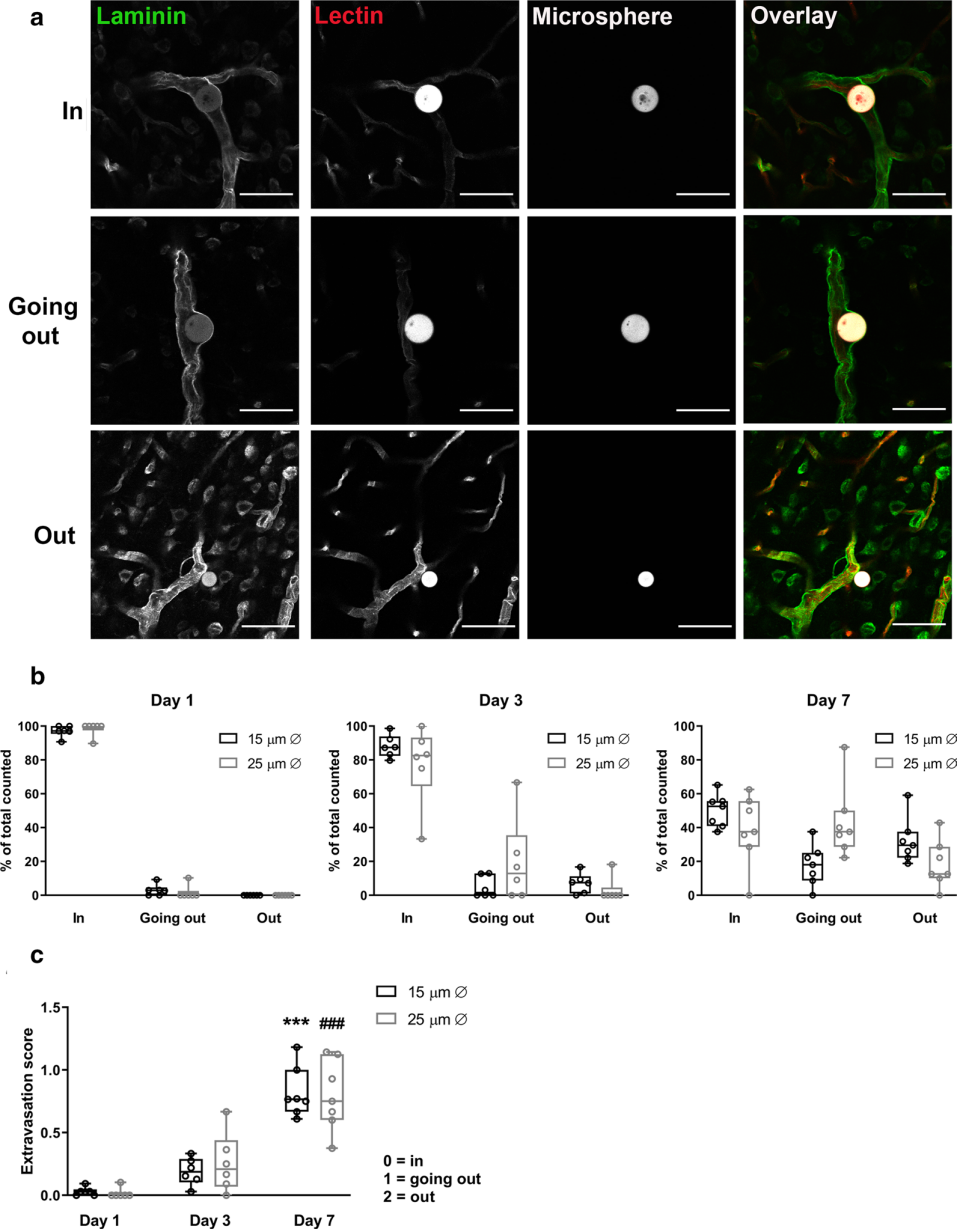
Microsphere extravasation in vivo is independent of size. **a** Examples of microspheres (white) scored as “in” (top panel), “going out” (middle panel) and “out” (bottom panel) in coronal brain sections stained postmortem for laminin (green) and i.v. lectin (red) before killing. Scale bar = 50 µm. **b** Quantification of microsphere extravasation at D1, 3 and 7 for 15 µm (black boxes) and 25 µm microspheres (grey boxes). N = 6–7 animals per time point. Data are depicted as median and IQR (min–max). **c** An extravasation score between 0 and 2 was calculated per rat to determine whether extravasation rate was dependent on time point and microsphere size (“in” was weighed as 0, “going out” as 1 and “out” as 2). N = 6–7 animals per time point. Data are depicted as median and IQR (min–max). ***P < 0.001, 15 µm D1 versus D7; ###*P* < 0.001, 20–30 µm D1 versus D7, Kruskal–Wallis with Dunn’s multiple comparison test

Based on staining for the extracellular matrix component laminin (Fig. 3a, Additional file 7: Video S2), we scored 15 and 25 µm microspheres as being inside a vessel (“in”); in the process of extravasation (“going out”/extraluminal); or outside of the vessel lumen and the extracellular matrix (“out”/parenchymal; see “Methods” section). Virtually all microspheres were inside the vessels at day (D)1. At D3, a small percentage of microspheres was in the process of extravasation, or had extravasated from the vessel. At D7, we found that for microspheres of 15 µm and 25 µm around half of the scored particles was either extraluminal or already parenchymal (Fig. 3b, Additional file 1: Supplementary Table S2).

We calculated an extravasation score, reflecting the average phase of extravasation per rat per day (see “Methods” section), for 15 µm and 25 µm microspheres. For both sizes of microspheres, the extravasation score was significantly higher at D7 compared to D1 (Fig. 3c; *P* = 0.0004 for 15 µm and *P* = 0.0007 for 25 µm microspheres, respectively). However, there was no effect of microsphere size on extravasation score (Fig. 3c, *P* = 0.8339, two-way RM ANOVA), suggesting that extravasation rate is similar for microspheres of 15 and 25 µm.

**Fig. 4:**
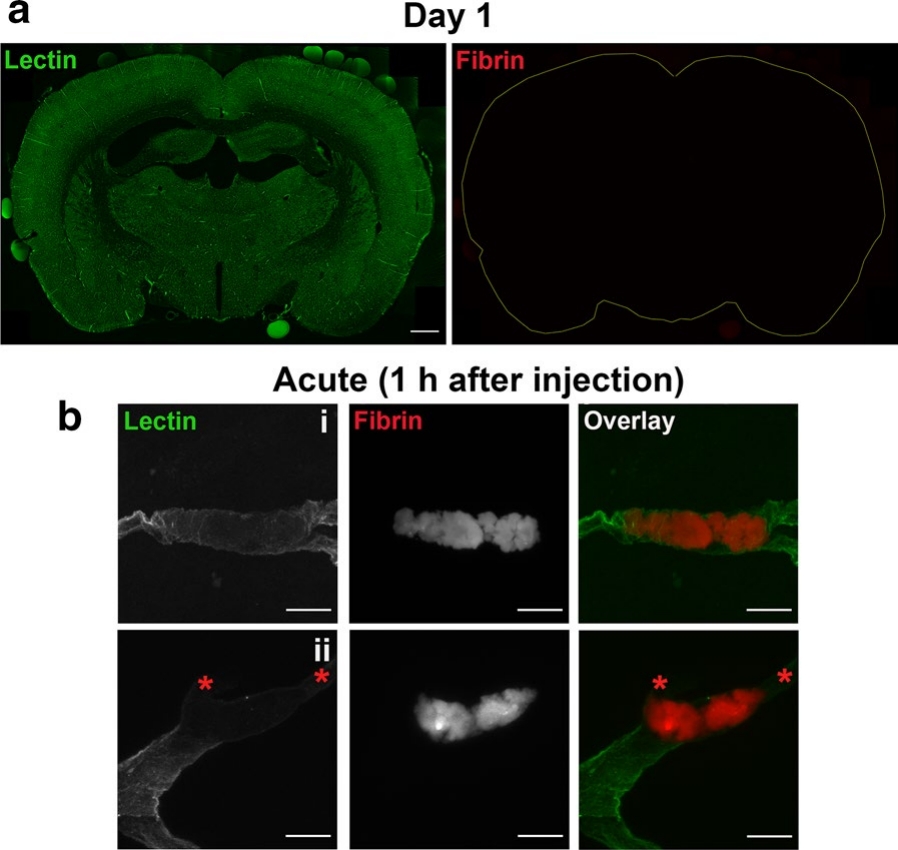
Microembolization with fibrin clots in vivo. **a** Fibrin clots (red) were not found in the brain vasculature (lectin; green), nor in the brain parenchyma at D1 after embolization. Scale bar = 1 mm. **b** Example (maximum intensity projections) of fibrin clots in the brain vasculature in an acute experiment, i.e. the rat was killed 1 h after injection with fibrin clots. (i) Fibrin clot (red) with lectin perfusion (green), and (ii) a fibrin clot occluding the vessel and hampering lectin perfusion. Scale bar = 10 µm

Lastly, since embolus composition may affect extravasation rate, we injected fluorescently-labeled fibrin clots ranging from ~ 10 to 40 µm and killed the rats at D1 (n = 4). To our surprise, we did not detect any fibrin clots or fragments inside the vessels or the brain parenchyma (Fig. 4a), nor did we find any fibrin in the retina, choroid plexus of the eye or the lungs (data not shown). In order to ascertain that the injections were successful, we added microspheres to the fibrin suspension and observed microspheres in the brain, but not fibrin clots. In contrast, we did detect fibrin clots occluding microvessels at 1 h after injection (Fig. 4b and Additional file 8: Video S3), ruling out a technical problem with fluorescent fibrin clots entering the brain. This suggests that fibrin clots may be processed within 24 h by the brain after intra-carotid injection, yet precludes the use of these emboli to further study angiophagy in our model.

### Microsphere extravasation is associated with vascular perfusion in vivo

**Fig. 5:**
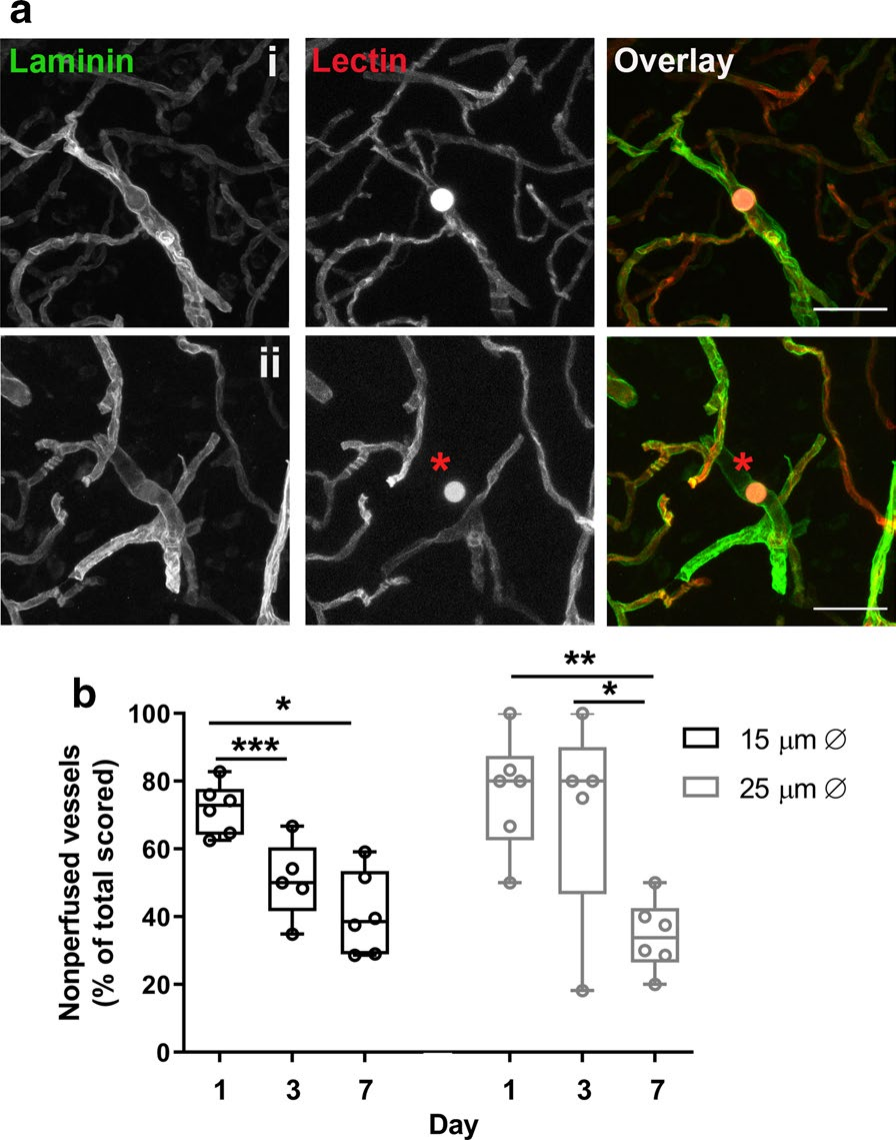
Quantification of perfused vessels after microembolization in vivo. **a** Example (maximum intensity projections) of (i) a microsphere (white) with lectin perfusion despite the presence of a microsphere, and (ii) a microsphere causing vascular nonperfusion (asterisk) using i.v. lectin (red) at the day of killing and postmortem laminin staining (green). Scale bar = 50 µm. **b** Quantification of vascular nonperfusion at D1, 3 and 7 for 15 µm (black boxes) and 25 µm microspheres (grey boxes). Three animals were excluded from perfusion analysis because the i.v. lectin injection failed, resulting in n = 5–6 animals per time point. Data are depicted as median and IQR (min–max). *P < 0.05, **P < 0.01, ***P < 0.001, one-way ANOVA with Tukey–Kramer’s test for multiple comparisons

Using an intravenous lectin injection on the day of killing and a postmortem laminin staining, we calculated the percentage of nonperfused vessels after microembolization with microspheres in rats. Figure 5a shows two examples of a vessel occluded by a microsphere enveloped by extracellular matrix (laminin): one with lectin perfusion despite the presence of a microsphere, and one lacking lectin signal, suggesting there was no vascular perfusion. Microembolization resulted in vascular nonperfusion in 72.9% and 80.0% of the scored vessels at D1 for 15 µm and 25 µm microspheres, respectively (IQR 64.2–77.7% for 15 µm and 62.5–87.5% for 25 µm; Fig. 5b). For both sizes of microspheres, the percentage of nonperfused vessels decreased significantly over time, with only 38.5% and 33.8% of nonperfusion at D7, respectively (IQR 28.9–53.5%; *P* = 0.0004, vs. D1 for 15 µm and IQR 26.5–42.5%; *P* = 0.0075, vs. D1 for 25 µm). In Table 1, the scored microspheres are categorized according to their extravasation status per day per size, and further classified into “perfused” or “nonperfused”. Using a Chi square test, we found that extravasation status and perfusion were strongly associated variables at all time points, showing that microsphere extravasation is associated with vessel reperfusion.

### Microspheres induce BBB opening in vivo

**Fig. 6:**
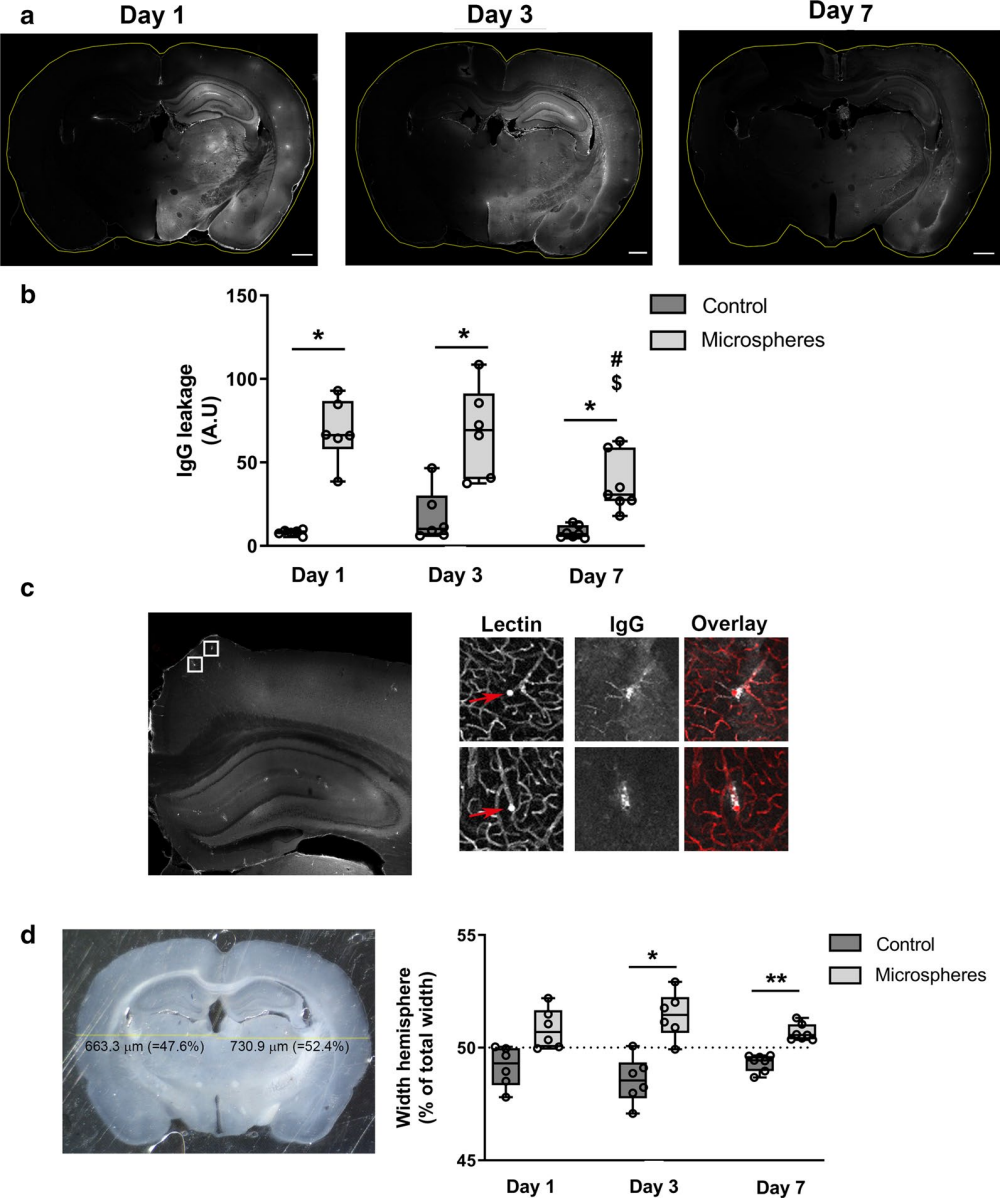
Microspheres induce BBB opening in vivo. **a** There was widespread IgG extravasation (white) throughout the injected hemisphere, whereas this was not the case in the control hemisphere. Scale bar = 1 mm. **b** Quantification of IgG signal intensity at D1, 3 and 7 in the control (dark grey) and injected (light grey) hemispheres. N = 6–7 animals per time point. Data are depicted as median and IQR (min–max). *P < 0.05, between hemispheres, Wilcoxon matched-pairs signed rank test; ^#^,^$^*P* < 0.05, D1 versus D7 and D3 versus D7, Kruskal–Wallis with Dunn’s multiple comparison test. **c** Accumulation of IgG was occasionally observed at places where microspheres had lodged (boxed regions in left overview image are enlarged in the right panel, microspheres are indicated by red arrows). **d** Width of control and treated hemispheres were measured in ex vivo coronal sections and quantification demonstrated that the treated hemisphere (light grey) expanded at the expense of the control hemisphere (dark grey). N = 6–7 animals per time point. Data are depicted as median and IQR (min–max). *P < 0.05, **P < 0.01, between hemispheres, paired t test

It takes several days for polystyrene microspheres to be extruded from the cerebral microvasculature (Fig. 3). The microsphere extravasation process itself, or the lack of perfusion of the occluded vessels, may lead to BBB disruption. Indeed, we observed widespread extravasation of IgG in the hemisphere in which we injected microspheres (Fig. 6a), indicative of a loss of BBB integrity. IgG signal intensity was significantly higher at all time points in the hemisphere injected with microspheres as compared to the control hemisphere (Fig. 6b). Signal intensity had decreased at D7, compared to D1 (*P* = 0.0279) and D3 (*P* = 0.0279), indicating that BBB opening may be temporary. IgG extravasation was observed throughout the entire hemisphere, yet with occasional accumulation at places where microspheres had lodged (Fig. 6c). Next, we measured the width of control and injected hemispheres in ex vivo coronal brain sections and found that the extensive BBB opening was accompanied by a midline shift, where the injected hemisphere expanded at the expense of the control hemisphere (Fig. 6d).

Finally, we observed mild reactive gliosis in the hemispheres injected with microspheres, demonstrated by immunostaining for reactive astrocytes with glial fibrillary acidic protein (GFAP) and activated microglia with ionized calcium-binding adaptor molecule 1 (Iba1). GFAP was expressed at higher levels in the hemisphere injected with microspheres as compared to the control hemisphere, peaking at D1 and D3, indicating the reactivity of surrounding astrocytes. Although expression appeared to have normalized at D7 (Additional file 9: Fig. S3A), quantification of the signal intensity showed sustained GFAP upregulation (Additional file 9: Fig. S3B). Iba1 expression increased in the hemisphere with microspheres at D3 and remained upregulated at D7 (Additional file 9: Fig. S3C, D). Moreover, microglia surrounding microspheres sometimes had a more amoeboid morphology, which is typical of an activated microglial phenotype, compared to the ramified morphology of quiescent microglia (Additional file 9: Fig. S3D). Taken together, microembolization and angiophagy are accompanied by transient BBB opening and mild gliosis.

## Discussion

Using a combination of in vitro and in vivo approaches, we here show in detail the extravasation of microspheres and fibrin clots. We found that processing of particles by endothelial cells is different for fibrin and microspheres, with uptake of fibrin clots through multiple F-actin-rich contact points and complete encapsulation by the cell. Microspheres on the other hand, are taken up by the endothelial cell through endothelial membrane protrusions and tunnel formation. The uptake of both microspheres and fibrin clots appears to occur anywhere in the cell, suggesting a preference for transcellular over paracellular transport. It was recently demonstrated that flow is required for endothelial cell remodeling and subsequent extravasation of tumor cells [10], in a mechanism similar to what we observed here. In that study, tumor cells were engulfed by endothelial cells which showed increased protrusions when kept under flow, yet this engulfment did not take place under static conditions [10]. In our hands, fibrin engulfment takes place under static conditions as shown by live cell imaging and immunofluorescence staining, and even inert polystyrene microspheres induce endothelial cell remodeling in the absence of flow. Thus, we show that flow is not a prerequisite for fibrin and microsphere uptake—in line with lack of flow in an occluded vessel in vivo—although it may very well enhance this process. In addition, polystyrene microspheres lack specific affinity to endothelial cells, and thus, microsphere uptake is independent of receptor-binding. Collectively and in line with previous studies [10, 12, 16], our results show that embolus uptake, transcellular transport and extravasation is associated with active remodeling of the endothelium.

In our rat model, we found that microembolization was accompanied by IgG extravasation and the affected hemisphere was enlarged at the expense of the control hemisphere, which is suggestive of edema. BBB opening was most prominent at early time points, when the large majority of microspheres occluded the vessels. Whether it is the vessel occlusion, or the process of angiophagy that is causing BBB opening is not clear from this study, but we suggest that BBB opening is temporary and will resolve once angiophagy proceeds and all microspheres have extravasated, as was shown previously [27]. Moreover, we observed mild reactive gliosis and activated microglia in the embolized hemisphere. Upon an insult, ramified microglia can transform into an amoeboid form and have the potential to become phagocytic [8]. The timing of microglial activation coincided with the first signs of microsphere extravasation, and thus may have been induced by the presence of foreign material in the brain parenchyma. Although we found no signs of microglial uptake of microspheres, it may very well be that microglia, or other perivascular cells such as pericytes, are involved in the post-extravasation degradation of more natural occurring microparticles on the tissue side [16].

Recently, it was demonstrated that cortical capillaries quite regularly undergo spontaneous obstruction [21], by what may be leukocytes or red blood cells [7]. To mimic such capillary obstructions and investigate what happens to occluded capillaries, microspheres of 4 µm were injected through the tail vein. In this study, the authors showed that over two-third is cleared by dislodgement of the microsphere back into the circulation, and only 2% through angiophagy [21]. However, microspheres of 4 µm can pass through the smallest capillaries [2], unlike microspheres sized > 15 µm as used in the present study, which occlude arterioles. Thus, small obstructions such as seen by Reeson et al. [21] may be cleared by dislodgment before angiophagy can take place.

Studies using optical methods such as focal photothrombosis to occlude single vessels [18, 23] showed that single capillary occlusions did not lead to detectable tissue damage, consistent with an interconnected microvascular network where flow loss can be compensated for by anastomosing vascular branches [23]. Although in our model we could not discriminate between what was proximal and distal from the microsphere, the fact that even at D1 a portion (about 20–30%) of the scored vessels had lectin perfusion despite the presence of an occluding microsphere, is in line with the idea that flow loss in the capillary bed may be partly compensated for by interconnected capillaries. However, we show that microvascular perfusion improves significantly over time and is strongly associated with extravasation status, with restored lectin perfusion in the vast majority of cases where microspheres have undergone or are in the process of extravasation. This argues for the importance of angiophagy in microvascular perfusion, and it being a protective mechanism of the brain, at times when collateral flow and enzymatic degradation are insufficient.

A limitation of our study is that we were unable to capture extravasation of fibrin clots in vivo. Whereas fibrin clots were found to occlude vessels and hamper lectin perfusion in an acute experimental setting (*i.e.* within 1 h after fibrin injection, Fig. 4, Additional file 8: Video S3), we could not detect any fibrin in the brain at 1 day after surgery, indicating that fibrin clots are processed rapidly and efficiently in rats. This was surprising, as studies in mice reported the presence of fibrin clots in the circulation or brain parenchyma until 8 days after injection [12, 16]. In the rat brain, the clots likely have undergone either intraluminal degradation or rapid angiophagy, followed by interstitial degradation. Previously, it was demonstrated in mice with in vivo two-photon imaging that both processes take place simultaneously, but that efficacy of enzymatic degradation decreased dramatically after the first 3 h after embolization, leaving the brain still with a substantial microembolic burden [12]. Similarly, another study found that only 40 to 50% of fibrin clots dissolved spontaneously in the mouse cerebral vasculature within 3 h, and this was paralleled with a sustained reduction in cerebral blood flow [1]. Therefore, we hypothesize that fibrin clots that have escaped enzymatic degradation were engulfed by the endothelium, extruded on the abluminal side and degraded in the interstitial space. This hypothesis is strengthened by several observations. Firstly, we did not find any evidence that particles that have been taken up are released back into the circulation (or the apical side in vitro). Since in vitro culture on glass coverslips is a limiting factor for the cells to extrude internalized particles as large as our fibrin clots or microspheres on the basolateral side, we found instead that both fibrin clots and microspheres are retained intracellularly (Additional file 5: Video 1, Additional file 6: Video 2). This internalization of fibrin took place within minutes, and in vivo, would hamper accessibility to blood-borne proteolytic enzymes, as was also suggested by Grutzendler et al. (2014) [12]. Therefore, it would be a feasible scenario that in vivo, the internalized particles are taken up and extruded basolaterally, as is the case with microspheres. Secondly, fibrin clots are not degraded by endothelial cells (Additional file 6: Video 2, Fig. 1C). However, endothelial cells and perivascular cells such as pericytes produce proteolytic enzymes [13], which may enhance fibrinolysis once the fibrin clots have been extruded. In fact, it was suggested that angiophagy is mediated by matrix metalloproteinases [16], which are also implicated in plasminogen-independent degradation of fibrin [14]. However, future studies are needed to thoroughly address the involvement of angiophagy in clearing of microthrombi that escaped enzymatic degradation, e.g. in models where fibrinolysis is impaired.

## Conclusions

Taken together, although angiophagy was associated with endothelial remodeling, temporary BBB opening and mild gliosis, our data suggest that it represents an important auxiliary mechanism of the cerebral vasculature to process emboli that cause widespread vessel occlusion. Considering that even inert, non-degradable microspheres are taken up by the endothelium, we believe that angiophagy takes place independent of receptor-binding, and embolic material of any source can be taken up and extruded by the microvessels. Thus, delineating its dynamics and underlying mechanisms is important and may lead to future interventions promoting angiophagy in the brain, reducing local hypoxia, edema and loss of function following an embolic event.

## Supplementary information

**Additional file 1**. Supplementary information: Supplementary Materials and Methods, Table S1, Table S2, Supplementary Figure and Video legends

**Additional file 2: Figure S1**. Size distribution of fibrin particles

**Additional file 3: Video S1**. Three-dimensional rendering of a microsphere (left panel) and fibrin clot (right panel) being taken up by HUVECs. Cells were stained for F-actin (red), VE-cadherin (green) and nuclei (DAPI, blue). Microsphere and fibrin clot are white. Scale bar = 20 µm

**Additional file 4: Figure S2**. Microspheres are taken up by hCMEC/D3 cells. **(A)** XZ and YZ orthogonal view of a z stack shows a cup structure of phalloidin (F-actin; red) surrounding the microsphere (white; in XZ and YZ depicted with dashed line), and F-actin- and VE-cadherin-positive “caps” on top of the microsphere. Different z planes are shown in i, ii and iii. Note the F-actin and VE-cadherin (green) surrounding the microsphere in ii, and the cap on top of the microsphere in iii. Scale bar = 10 µm. **(B)** Quantification of signal intensity for F-actin, VE = cadherin and microsphere in the z direction shows a peak in signal intensity after the microsphere, which is the cap structure on top of the microsphere. Light-colored lines are signal intensity in control location, *i.e.* of a region where no microsphere was bound. Signal intensity was quantified from 2-4 images averaged from n = 3 independent experiments. Data are depicted as mean ± S.D. (dashed lines). **(C)** Three-dimensional rendering of a fibrin clot, encapsulated by the cytoskeleton. Left panel is the view from below the cellular monolayer, right panel is the view from above the monolayer. Right panel shows that the fibrin clot is taken up by two cells, demonstrated by the two cell nuclei (DAPI; blue). Scale bar = 10 µm

**Additional file 5:Video 1**. Live cell imaging showing microsphere uptake by HUVECs. HUVECs were transduced with lentiviral LifeAct (green) to visualize the cytoskeleton and images were taken every 2 min over 8 h in xy. Left panel: LifeAct (green) and a 15 µm microsphere (white; coming in from the left) and fibrin clot (white; coming in from the right). Right panel: only LifeAct signal in grey. The endothelium generates protrusions and a F-actin rich cup structure surrounding the microsphere, occurring after ± 4 h after microsphere addition (red arrow indicates the endothelial cup)

**Additional file 6: Video 2**. Live cell imaging showing fibrin clot uptake by HUVECs. HUVECs were transduced with lentiviral LifeAct (green) to visualize the cytoskeleton and images were taken every minute over 8 h in xy. Left panel: LifeAct (green) and fibrin clot (white). Right panel: only LifeAct signal in grey. Fibrin clots are taken up by the cell through multiple F-actin-rich points of contact (open arrow) and, once taken up in the cell, leave an imprint in the F-actin cytoskeleton (filled arrow). Fibrin clots are retained by the cell, despite cell locomotion and division, and are not degraded intracellularly

**Additional file 7: Video S2**. Three-dimensional rendering of examples of a microsphere inside a vessel (“in”; left panel), microsphere inside a vessel but with a laminin bulge and restored perfusion (“going out”/extraluminal; middle panel) and outside the vessel lumen and extracellular matrix (“out”/parenchymal; right panel). Laminin (green) and microsphere (white). Scale bar = 50 µm

**Additional file 8:Video S3**. Three-dimensional view of a fibrin clot (red) occluding a cerebral vessel visualized by i.v. lectin perfusion (green) prior to killing in an acute experimental setting (*i.e.* animal was killed within 1 h after embolization surgery, n = 1). Note the lack of lectin perfusion distal from the fibrin clot. Scale bar = 20 µm

**Additional file 9: Figure S3**. Microspheres induce mild reactive gliosis in vivo. **(A)** GFAP staining (green) was increased in the treated hemisphere. Scale bar = 1 mm. **(B)** Quantification of GFAP signal intensity at D1, D3 and 7 in the control (dark grey) and injected (light grey) hemispheres. N = 6-7 animals per time point. Data are depicted as median and IQR (min – max). *P < 0.05, between hemispheres, Wilcoxon matched-pairs signed rank test. **(C)** Iba1 staining (white) was increased in the treated hemisphere. Scale bar = 1 mm. **(D)** Quantification of Iba1 signal intensity at D1, D3 and 7 in the control (dark grey) and injected (light grey) hemispheres. N = 6-7 animals per time point. Data are depicted as median and IQR (min – max). *P < 0.05, between hemispheres, Wilcoxon matched-pairs signed rank test. Reactive microglia (Iba1; white) were observed surrounding microspheres (red) with a changed morphology (from ramified in the control hemisphere to amoeboid surrounding microspheres). Scale bar = 50 µm

## Abbreviations

BBB: Blood-brain barrier
CCA: Common carotid artery
COVID-19: Coronavirus Disease 2019
ECA: External carotid artery
ECIS: Electric cell-substrate impedance sensing
F-actin: Filamentous actin
GFAP: Glial fibrillary acidic protein
hCMEC/D3: Human cerebral microvascular endothelial cell line
HUVECs: Human umbilical vein endothelial cells
Iba1: Ionized calcium-binding adapter molecule 1
ICA: Internal carotid artery
IgG: Immunoglobulin G
i.p.: Intraperitoneal
NA: Numerical aperture
PBS: Phosphate buffered saline
VE-cadherin: Vascular endothelial cadherin

## Availability of data and material

The datasets used and analysed during the current study are available from the corresponding author on reasonable request.
