# Supplementary material for: Microembolus clearance through angiophagy is an auxiliary mechanism preserving tissue perfusion in the rat brain
Source: Acta Neuropathol Commun. 2020 Nov 17;8:195. doi: 10.1186/s40478-020-01071-9 (PMC7671188; doi:10.1186/s40478-020-01071-9)
Supplement: Supplementary file 1 — Additional file 1. Supplementary information: Supplementary Materials and Methods, Table S1, Table S2, Supplementary Figure and Video legends [file 40478_2020_1071_MOESM1_ESM.docx]

**SUPPLEMENTARY INFORMATION**

**Microembolus clearance through angiophagy is an auxiliary mechanism preserving tissue perfusion in the rat brain**

Anne-Eva van der Wijk*, Theodosia Georgakopoulou*, Jisca Majolée, Jan S.M. van Bezu, Miesje M. van der Stoel, Bert J. van het Hof, Helga E. de Vries, Stephan Huveneers, Peter L. Hordijk, Erik N.T.P. Bakker, Ed vanBavel

*Shared first authorship

**Supplementary Materials and Methods**

*Fibrin clots*

Fibrin clots were made as described by Lam et al. (2010) [1]. In short, fibrinogen from bovine plasma (Cat #F8630, Sigma-Aldrich; 100 mg/ml) was dissolved in PBS and conjugated with Texas Red succinimidyl ester (Cat #T6134, ThermoFisher; 5 mg/ml in DMSO) for 1 h at room temperature. Next, bovine thrombin (500 U/ml in 0.1% BSA; Cat #112374, Merck Millipore) was added to the fibrinogen (62.5 U/ml end concentration), immediately vortexed and incubated at 40 ºC overnight. The resulting fibrin clot was fragmented into small pieces by crude mechanical disruption and sonication, washed with ethanol to remove unbound ester, resuspended in PBS and filtered through cell strainers to eliminate debris and small particles, in order to get fibrin clots in the 10-40 µm range (Figure S1, Additional File 2).

*Immunofluorescence staining of cells*

Cells were carefully washed in PBS and permeabilized with 0.2% TritonX-100 for 3 min, washed in PBS and blocked with 1% human serum albumin (HSA) for 1 h. Next, cells were incubated in rabbit anti-VE cadherin (D87F2) XP® (1:400, Cat #2500, Cell Signaling, Danvers, MA) diluted in 1% HSA for 2 h. After three wash steps (3 times 5 min in PBS), cells were incubated for 1 h in goat anti-rabbit Alexa Fluor 488 (1:100, Invitrogen), a phalloidin probe (Acti-stain 555 phalloidin, 1:200, Cat #PHDH1, Cytoskeleton, Heerhugowaard, The Netherlands) and 4’,6-diamidino-2-phenylindole (DAPI; 1:500) for nuclear staining diluted in 1% HSA. Cells were washed (three times 5 min in PBS) and coverslips were mounted with Mowiol® 4-88/DABCO solution (Sigma-Aldrich). All staining procedures were performed at room temperature.

*Electric cell-substrate impedance sensing (ECIS)*

Endothelial barrier function was assessed with electric cell-substrate impedance sensing (ECIS; Applied BioPhysics, Inc., Troy, NY). HUVECs were isolated as described previously [2] and seeded on 1% gelatin-coated gold electrode arrays (96W10idf; Applied BioPhysics). Impedance was measured during monolayer formation at 4 kHz with ECIS software (Applied BioPhysics). After formation of a stable endothelial barrier, microparticles were added to the cell medium in different concentrations (1600, 3200 or 6400 microspheres per well (surface area 0.32 cm^2^) and 450, 900 or 1800 fibrin particles per well (surface area 0.32 cm^2^) and impedance was measured for another 24 h. For microspheres, a 2% BSA control was included, in addition to control wells where only PBS was added to the medium. Five independent experiments were done in triplo.

*Rat brain preparation and immunofluorescence staining*

The brain was removed and further processed as described previously [3]. Brain sections (100 µm thick) were stained as described previously [3], with the following adjustments: a different blocking buffer was used (5% normal goat serum, 2% Triton X-100 and 0.2% NaN3 in PBS), all steps were done at room temperature and brain sections were incubated in secondary antibody for 2 h. The following antibodies were used: (primary) rabbit polyclonal anti-laminin antibody (diluted 1:500, Cat #L9393; Sigma-Aldrich, Zwijndrecht, The Netherlands), mouse monoclonal anti-GFAP antibody (diluted 1:200, Cat #4650-0309, Bio-Rad, Oxford, UK), rabbit polyclonal anti-Iba 1 antibody (diluted 1:1000, Cat #019-19741, Wako, Neuss, Germany) and (secondary) goat-anti-mouse Cy5 or goat-anti-rabbit Alexa 488; diluted 1꞉200) diluted in blocking buffer. The IgG staining was done by incubating brain sections with a goat anti-rat-IgG conjugated to Cy5 (diluted 1:250, Cat #A21208, ThermoFisher, Landsmeer, The Netherlands) overnight at room temperature. Specificity of the staining was checked by excluding the primary antibody.

**Supplementary Tables**

**Supplementary Table S1. Microsphere distribution**

| Microsphere Ø: | 15 µm | 25 µm | 50 µm |
| --- | --- | --- | --- |
| *Injected (#)* | 25,000 | 5,500 | 625 |
| *% of total* | 80.3 | 17.7 | 2.0 |
| *Scored per animal (#)* | 40 ± 21 | 9 ± 5 | 0 ± 1 |
| *Scored per animal (%)* | 79.2 ± 7.9 | 20.2 ± 7.8 | 0.6 ± 1.1 |

**n=19 rats, total of 937 microspheres. mean ± s.d.**

**Supplementary Table S2. Microsphere extravasation status**

|  | In (#) | Going out (#) | Out (#) | Total scored (#) |
| --- | --- | --- | --- | --- |
| Day 1 (n=6) |  |  |  |  |
| *15 µm* | 187 | 7 | 0 | 194 |
| *25 µm* | 54 | 4 | 0 | 57 |
| Day 3 (n=6) |  |  |  |  |
| *15 µm* | 250 | 17 | 15 | 282 |
| *25 µm* | 52 | 6 | 2 | 60 |
| Day 7 (n=7) |  |  |  |  |
| *15 µm* | 112 | 39 | 75 | 226 |
| *25 µm* | 28 | 31 | 13 | 72 |

**Supplementary Figure and Video legends**

*Video S1.* Three-dimensional rendering of a microsphere (left panel) and fibrin clot (right panel) being taken up by HUVECs. Cells were stained for F-actin (red), VE-cadherin (green) and nuclei (DAPI, blue). Microsphere and fibrin clot are white. Scale bar = 20 µm.

*Video S2.* Three-dimensional rendering of examples of a microsphere inside a vessel (“in”; left panel), microsphere inside a vessel but with a laminin bulge and restored perfusion (“going out” / extraluminal; middle panel) and outside the vessel lumen and extracellular matrix (“out” / parenchymal; right panel). Laminin (green) and microsphere (white). Scale bar = 50 µm.

*Video S3.* Three-dimensional view of a fibrin clot (red) occluding a cerebral vessel visualized by i.v. lectin perfusion (green) prior to killing in an acute experimental setting (*i.e.* animal was killed within 1 h after embolization surgery, n=1). Note the lack of lectin perfusion distal from the fibrin clot. Scale bar = 20 µm.

*Figure S1*. Size distribution of fibrin particles.

*Figure S2.* Microspheres are taken up by hCMEC/D3 cells. **(A)** XZ and YZ orthogonal view of a z stack shows a cup structure of phalloidin (F-actin; red) surrounding the microsphere (white; in XZ and YZ depicted with dashed line), and F-actin- and VE-cadherin-positive “caps” on top of the microsphere. Different z planes are shown in i, ii and iii. Note the F-actin and VE-cadherin (green) surrounding the microsphere in ii, and the cap on top of the microsphere in iii. Scale bar = 10 µm. **(B)** Quantification of signal intensity for F-actin, VE=cadherin and microsphere in the z direction shows a peak in signal intensity after the microsphere, which is the cap structure on top of the microsphere. Light-colored lines are signal intensity in control location, *i.e.* of a region where no microsphere was bound. Signal intensity was quantified from 2-4 images averaged from n = 3 independent experiments. Data are depicted as mean ± s.d. (dashed lines). **(C)** Three-dimensional rendering of a fibrin clot, encapsulated by the cytoskeleton. Left panel is the view from below the cellular monolayer, right panel is the view from above the monolayer. Right panel shows that the fibrin clot is taken up by two cells, demonstrated by the two cell nuclei (DAPI; blue). Scale bar = 10 µm.

*Figure S3.* Microspheres induce mild reactive gliosis *in vivo*. **(A)** GFAP staining (green) was increased in the treated hemisphere. Scale bar = 1 mm. **(B)** Quantification of GFAP signal intensity at D1, D3 and 7 in the control (dark grey) and injected (light grey) hemispheres. N=6-7 animals per time point. Data are depicted as median and IQR (min – max). *P<0.05, between hemispheres, Wilcoxon matched-pairs signed rank test. **(C)** Iba1 staining (white) was increased in the treated hemisphere. Scale bar = 1 mm. **(D)** Quantification of Iba1 signal intensity at D1, D3 and 7 in the control (dark grey) and injected (light grey) hemispheres. N=6-7 animals per time point. Data are depicted as median and IQR (min – max). *P<0.05, between hemispheres, Wilcoxon matched-pairs signed rank test. Reactive microglia (Iba1; white) were observed surrounding microspheres (red) with a changed morphology (from ramified in the control hemisphere to amoeboid surrounding microspheres). Scale bar = 50 µm.

**References**

1 Lam CK, Yoo T, Hiner B, Liu Z, Grutzendler J (2010) Embolus extravasation is an alternative mechanism for cerebral microvascular recanalization. Nature 465: 478-482 Doi 10.1038/nature09001

2 Majolee J, Pronk MCA, Jim KK, van Bezu JSM, van der Sar AM, Hordijk PL, Kovacevic I (2019) CSN5 inhibition triggers inflammatory signaling and Rho/ROCK-dependent loss of endothelial integrity. Sci Rep 9: 8131 Doi 10.1038/s41598-019-44595-4

3 van der Wijk AE, Lachkar N, de Vos J, Grootemaat AE, van der Wel NN, Hordijk PL, Bakker E, vanBavel E (2019) Extravasation of Microspheres in a Rat Model of Silent Brain Infarcts. Stroke 50: 1590-1594 Doi 10.1161/STROKEAHA.119.024975
