# Supplementary figures and images for: Microembolus clearance through angiophagy is an auxiliary mechanism preserving tissue perfusion in the rat brain
Source: Acta Neuropathol Commun. 2020 Nov 17;8:195. doi: 10.1186/s40478-020-01071-9 (PMC7671188; doi:10.1186/s40478-020-01071-9)

Figure S1.

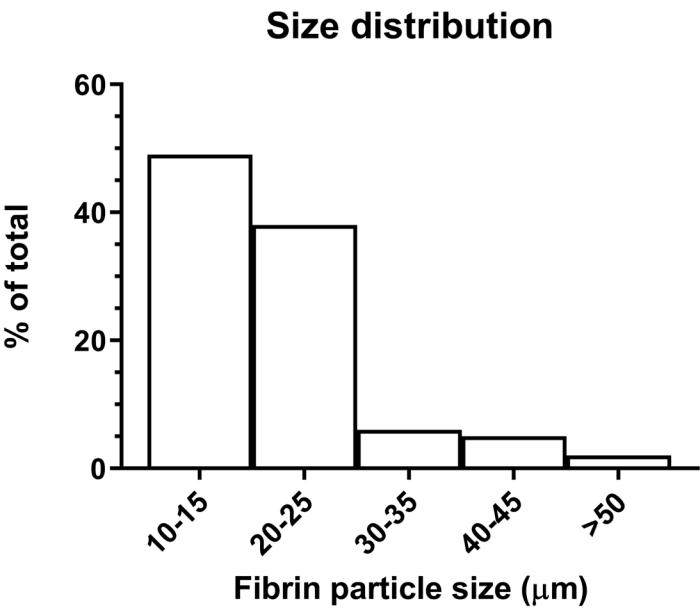

Supplement: Supplementary file 2 — Additional file 2: Figure S1. Size distribution of fibrin particles [file 40478_2020_1071_MOESM2_ESM.pdf]

**Figure S2.**

**A** hCMEC/D3 cells (4h)

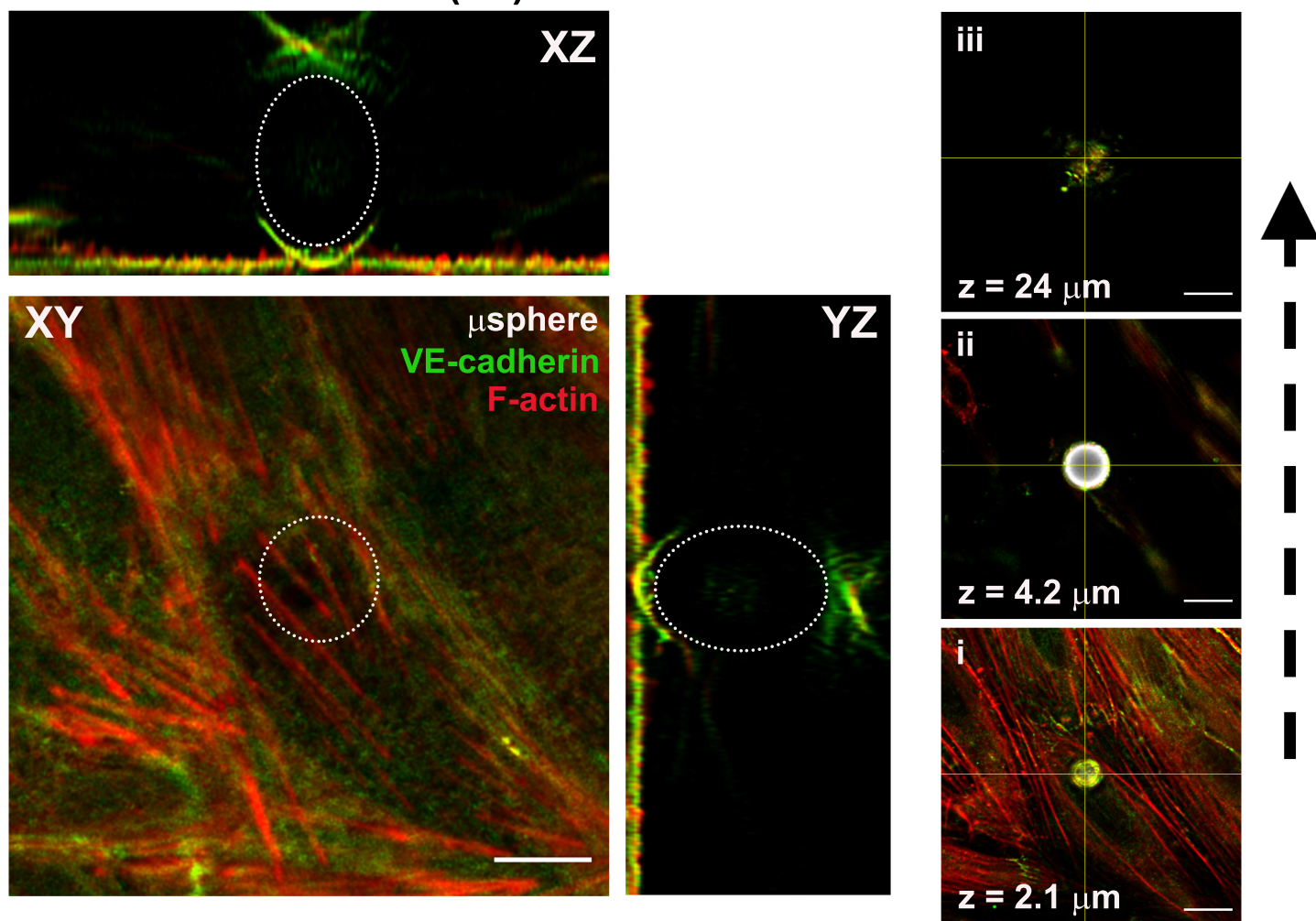

**B**

4h

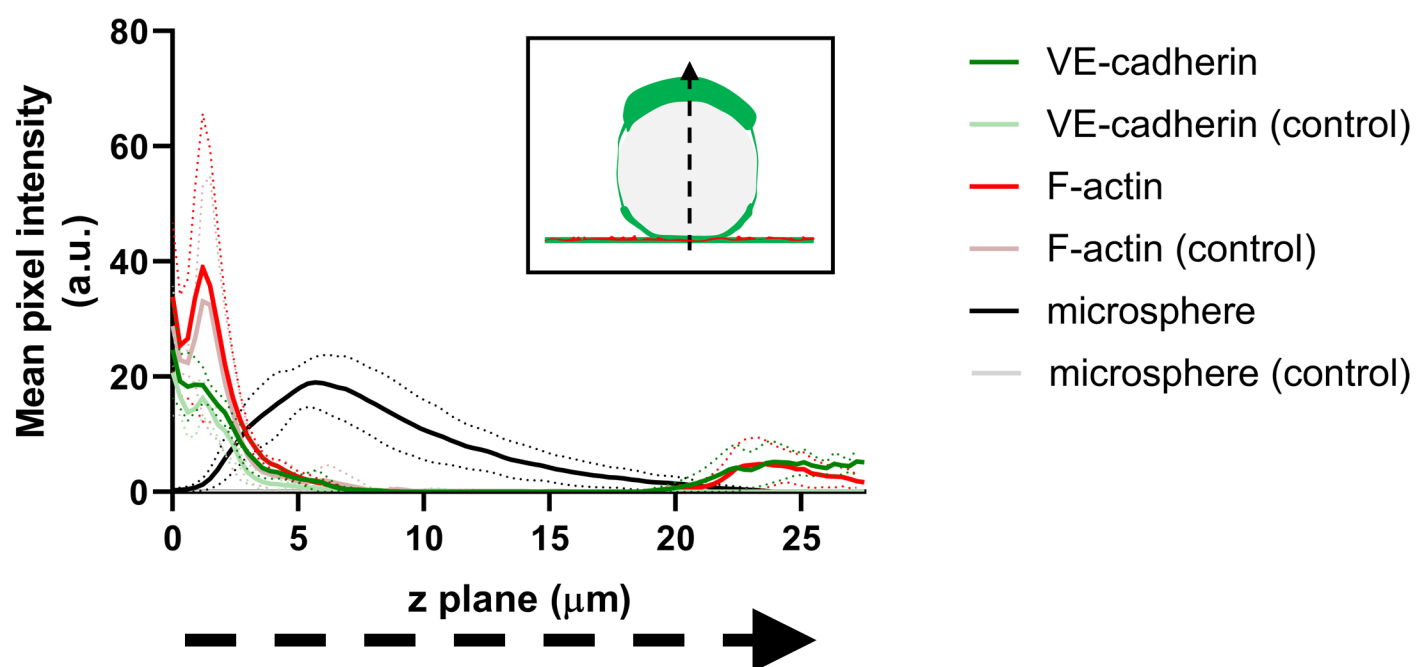

**C**

hCMEC/D3 cells (24h)

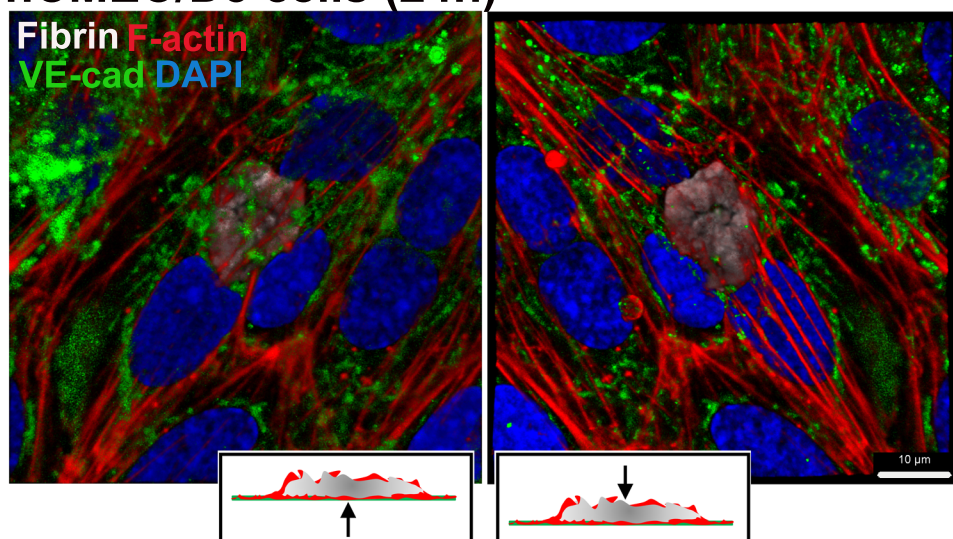

Supplement: Supplementary file 4 — Additional file 4: Figure S2. Microspheres are taken up by hCMEC/D3 cells. (A) XZ and YZ orthogonal view of a z stack shows a cup structure of phalloidin (F-actin; red) surrounding the microsphere (white; in XZ and YZ depicted with dashed line), and F-actin- and VE-cadherin-positive “caps” on top of the microsphere. Different z planes are shown in i, ii and iii. Note the F-actin and VE-cadherin (green) surrounding the microsphere in ii, and the cap on top of the microsphere in iii. Scale bar = 10 µm. (B) Quantification of signal intensity for F-actin, VE = cadherin and microsphere in the z direction shows a peak in signal intensity after the microsphere, which is the cap structure on top of the microsphere. Light-colored lines are signal intensity in control location, i.e. of a region where no microsphere was bound. Signal intensity was quantified from 2-4 images averaged from n = 3 independent experiments. Data are depicted as mean ± S.D. (dashed lines). (C) Three-dimensional rendering of a fibrin clot, encapsulated by the cytoskeleton. Left panel is the view from below the cellular monolayer, right panel is the view from above the monolayer. Right panel shows that the fibrin clot is taken up by two cells, demonstrated by the two cell nuclei (DAPI; blue). Scale bar = 10 µm [file 40478_2020_1071_MOESM4_ESM.pdf]

**Figure S3.**

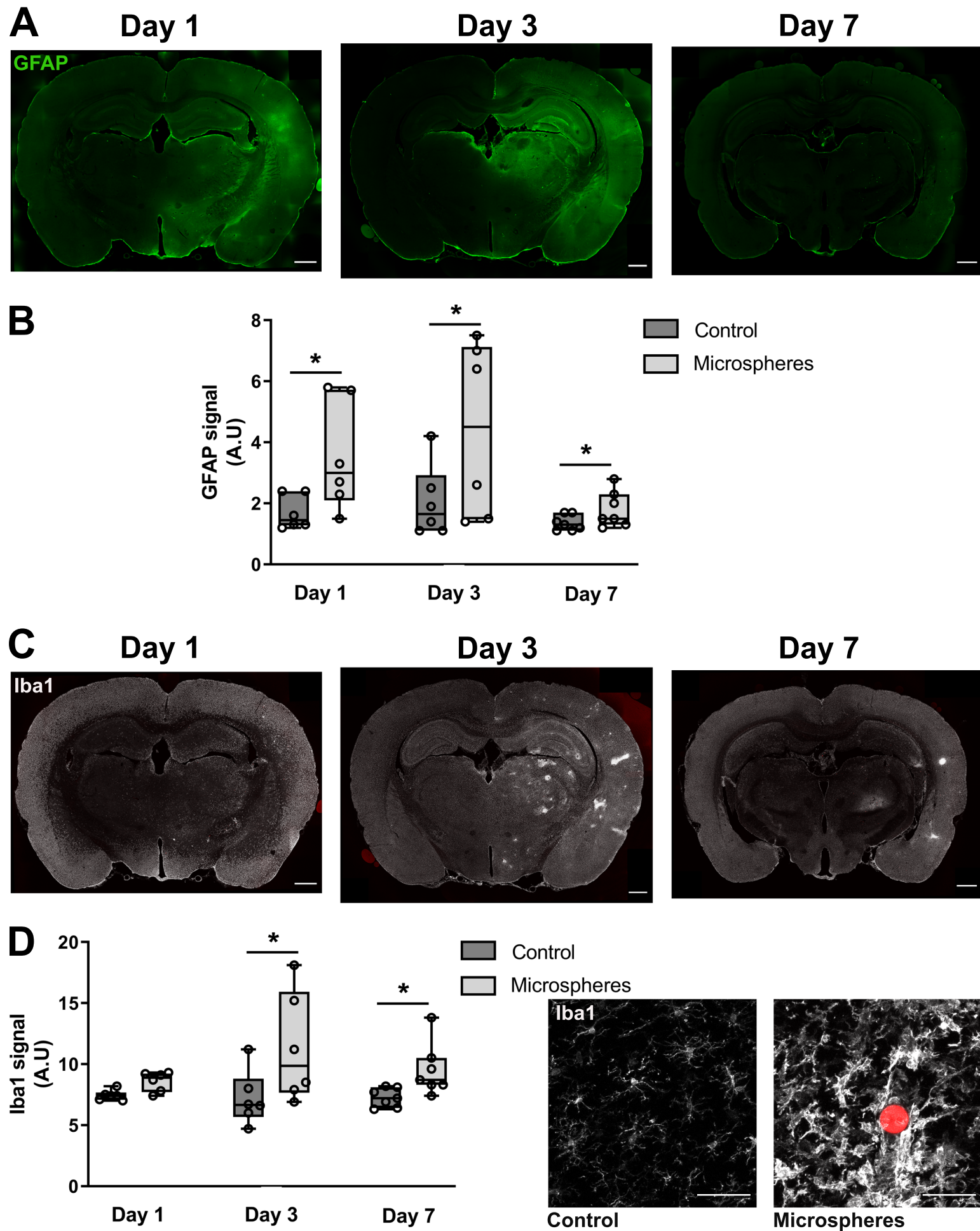

Supplement: Supplementary file 9 — Additional file 9: Figure S3. Microspheres induce mild reactive gliosis in vivo. (A) GFAP staining (green) was increased in the treated hemisphere. Scale bar = 1 mm. (B) Quantification of GFAP signal intensity at D1, D3 and 7 in the control (dark grey) and injected (light grey) hemispheres. N = 6-7 animals per time point. Data are depicted as median and IQR (min – max). *P < 0.05, between hemispheres, Wilcoxon matched-pairs signed rank test. (C) Iba1 staining (white) was increased in the treated hemisphere. Scale bar = 1 mm. (D) Quantification of Iba1 signal intensity at D1, D3 and 7 in the control (dark grey) and injected (light grey) hemispheres. N = 6-7 animals per time point. Data are depicted as median and IQR (min – max). *P < 0.05, between hemispheres, Wilcoxon matched-pairs signed rank test. Reactive microglia (Iba1; white) were observed surrounding microspheres (red) with a changed morphology (from ramified in the control hemisphere to amoeboid surrounding microspheres). Scale bar = 50 µm [file 40478_2020_1071_MOESM9_ESM.pdf]
